# *GAL1-SceI *directed site-specific genomic (gsSSG) mutagenesis: a method for precisely targeting point mutations in *S. cerevisiae*

**DOI:** 10.1186/1472-6750-11-120

**Published:** 2011-12-05

**Authors:** Sarah Piccirillo, Hsiao-Lin Wang, Thomas J Fisher, Saul M Honigberg

**Affiliations:** 1 School of Biological Sciences, University of Missouri-Kansas City, Kansas City MO 64110, USA; 2 Dept. of Mathematics and Statistics, University of Missouri-Kansas City, Kansas City MO 64110, USA

## Abstract

**Background:**

Precise targeted mutations are defined as targeted mutations that do not require the retention of other genetic changes, such as marker genes, near the mutation site. In the yeast, *S. cerevisiae*, there are several methods for introducing precise targeted mutations, all of which depend on inserting both a counter-selectable marker and DNA bearing the mutation. For example, the marker can first be inserted, and then replaced with either a long oligonucleotide carrying the mutation (*delitto perfetto*) or a PCR fragment synthesized with one primer containing the mutation (SSG mutagenesis).

**Results:**

A hybrid method for targeting precise mutation into the genomes uses PCR fragments as in SSG mutagenesis together with a CORE cassette devised for *delitto perfetto *that contains the homing endonuclease SceI. This method, termed gsSSG mutagenesis, is much more efficient than standard SSG mutagenesis, allowing replacements to be identified without extensive screening of isolates. In gsSSG, recombination between the PCR fragment and the genome occurs equally efficiently regardless of the size of the fragment or the distance between the fragment end and the site of marker insertion. In contrast, the efficiency of incorporating targeted mutations by this method increases as the distance between the mutation and the marker insertion site decreases.

**Conclusion:**

gsSSG is an efficient way of introducing precise mutations into the genome of *S. cerevisiae*. The frequency of incorporating the targeted mutation remains efficient at least as far as 460 bp from the insertion site meaning that a single insertion can be used to create many different mutants. The overall efficiency of gsSSG can be estimated based on the distance between the mutation and the marker insertion, and this efficiency can be maximized by limiting the number of untargeted mutations. Thus, a single insertion of marker genes plus homing endonuclease cassette can be used to efficiently introduce precise point mutations through a region of > 900 bp.

## Background

The budding yeast *S. cerevisiae *has been a premier genetic model organism for many years in large part because of the ability of yeast geneticists to delete targeted genes. This ability results from extraordinarily efficient homologous recombination between exogenous DNA fragments and the yeast genome. More recently it has also become possible to knockout or knockdown gene expression in many other species. However, *S. cerevisiae *remains one of the few organisms in which point mutations can be efficiently introduced into the genome that are both "targeted" and "precise". In this context, "targeted" means that a particular locus, for example a specific base pair or a short sequence, is designated for a specific genetic change. "Precise" means that no other changes are introduced into the final mutant besides the targeted mutation; for example, no marker genes or random mutations are present in the final mutant strain. Targeted precise mutations are particularly useful when the goal is to measure the effect of relatively small changes in the genome on phenotype.

Although it is typically quicker to introduce mutations into a plasmid-borne copy of a gene than into the genome, there are several advantages to making the mutation in the genome, particularly when measuring the effect of these mutations on gene expression. First, the packaging of DNA on chromosomes (i.e. the chromatin structure) can be different on plasmid vectors than on chromosomes, and these differences will sometimes affect gene expression [1-3]. Second, distant chromosomal elements affecting transcription in the endogenous gene may be absent from the limited region that can be cloned into a vector. Third, small differences in gene expression are difficult to assay reliably in plasmids because unavoidable random fluctuations in plasmid copy number can also affect transcript levels [4-6]. For all of these reasons, methods for efficiently introducing precise mutations into the genome can be extremely valuable for studying gene regulatory sequences.

The original method for making precise targeted mutations utilizes linearized plasmids that could be inserted into the genome and then removed [7]. This "pop-in/pop-out replacement" strategy typically utilizes a plasmid containing both a mutant allele and the *URA3 *marker. In the pop-in step, the plasmid is linearized at a site within the mutant allele and then placed inside a *ura3Δ *strain (transformation), at which point recombination takes place between the mutant allele and its genomic homolog. Strains that have the plasmid inserted into their genome are selected for on medium lacking uracil, and the site of insertion verified by molecular analysis. These strains have two tandem alleles present at the site of insertion separated by the plasmid and *URA3*. In the pop-out step, these strains are then exposed to the drug 5-fluoroorotic acid (FOA), which selects for rare isolates in which recombination between the two alleles results in loss of *URA3*.

A variation of the above method uses a PCR fragment containing a short direct repeat surrounding *URA3 *[8]. After this fragment is inserted, the marker can be excised as a result of recombination between the direct repeats. When these direct repeats contain a mutation, this mutation will then be precisely introduced into the genome. An updated version of this method, termed MIRAGE, adds an inverted repeat of the marker gene in order to increase the efficiency of marker excision [9]. These PCR fragment-base methods have the advantage of not requiring subcloning; for example, in MIRAGE the fragment introduced into the genome is constructed by in vitro ligation of two PCR fragments followed by gel purification of the product.

Another method that does not require subcloning is *delitto perfetto *(Italian slang for "perfect murder") [10]. Unlike the above methods, which require only a single transformation, *delitto perfetto *requires two sequential transformations. In the first transformation, a PCR fragment containing *URA3 *(and a second marker to increase the selection power) is inserted into the genome, directed to the genomic target site by approximately 60 bp of homology at each end of the PCR fragment. In the second transformation, the inserted markers are replaced by a long (> 80 bp) mostly double-strand oligonucleotide that spans the inserted markers and contains the targeted mutation. These recombinants are selected on FOA. Although *delitto perfetto *requires one more transformation than the above methods, the DNA fragments used do not require subsequent manipulation such as ligation or gel purification. As a result, *delitto perfetto *is particularly useful when the goal is to generate several mutants, each containing a different mutation, within the same 100-200 bp region of the genome, with the size of this region limited only by the size of the oligonucleotide that can be synthesized. A different oligonucleotide-based method, termed multiplex automated genome engineering (MAGE), has recently been developed to target precise mutations into *E. coli*. MAGE is particularly useful for targeting multiple precise mutation combinations throughout the genome [11].

An augmented version of *delitto perfetto *introduces a *GAL1*-promoter driven restriction enzyme gene, *Sce*I (*GAL1-Sce*I) into the genome in the first transformation (in addition to the marker genes). At the same time, an 18 bp SceI site absent elsewhere in the yeast genome is also introduced [12,13]. This cassette is here referred to as "CORE-GS." In this improved version of *delitto perfetto*, galactose is used to induce the *GAL1 *promoter prior to the second transformation, leading to expression of SceI and hence cleavage at the SceI site. This break increases the recombination efficiency 3-4 orders of magnitude relative to the original protocol.

**Figure 1:**
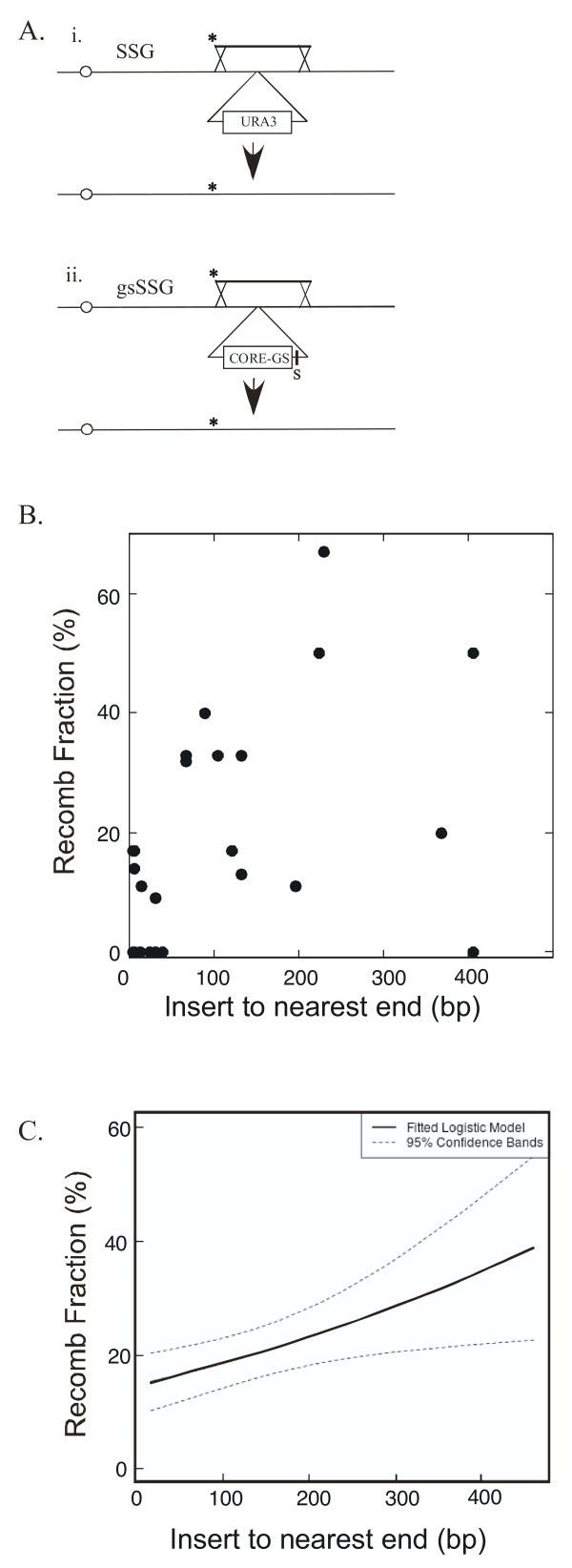
**Recombination between PCR fragment and genome leading to marker replacement**. A) Standard SSG mutagenesis (i) and gsSSG mutagenesis (ii). Diagrams show recombination between PCR fragment (top line) and chromosome (bottom line with centromere represented as open circle). Asterisk indicates the targeted mutation, and the "S" in (ii) indicates position of the SceI site, which is introduced on one of the primers. B) Relationship between the distance between marker insertion site and closest end of PCR fragment and recombinant fraction. Recombinant fraction, defined as the fraction of FOA^R ^isolates that derive from marker replacement is shown for standard SSG mutagenesis transformants with the nearest end at varying distances between the marker insertion site and the nearest fragment end. Only transformations where ≥ 5 FOA^R ^isolates were analyzed are included in this graph. C) Logistic regression analysis of the same data for SSG mutagenesis as in B) except that all data was included regardless of the number of isolates analyzed for a given transformation. The solid line represents the fitted logistic model, and the dashed line represents 95% confidence bands around the model.

A related method to *delitto perfetto *that also involves two sequential transformations inserts *URA3 *in the first step (as in *delitto perfetto*) and then replaces this marker with a PCR fragment [14]. In this method, termed site-specific genomic (SSG) mutagenesis, point mutations can be introduced using the primer at either end of the PCR fragment, so the region that can be mutagenized from a single marker insertion is larger than in *delitto perfetto *(Figure 1A). Variations on SSG mutagenesis have been used to introduce random mutations on the PCR fragment [14], and also to target precise small deletions, insertions, and allele substitutions into the genome [15].

In the current study, we examined the effect of the CORE-GS insertion on the efficiency of SSG mutagenesis, generating a "hybrid" method between *delitto perfetto *and SSG mutagenesis that we term "gsSSG mutagenesis". Using this hybrid protocol, we targeted mutations to over 30 sites within an approximately 700 bp region. By measuring the efficiency of incorporating mutations at these sites, we identified parameters that determine the success rate of gsSSG. Finally, we discuss the advantages and disadvantages of gsSSG relative to other methods for creating precise mutations.

## Results

### CORE-GS increases the efficiency of PCR-fragment insertion

The key step that limits the efficiency of SSG mutagenesis is the second transformation, and the success of this step depends on recombination between a PCR fragment containing a mutation and a region of the genome containing *URA3*, resulting in the replacement of *URA3 *with the PCR fragment (Figure 1A (i)). Because this recombination occurs at very low frequencies, the resulting *ura3Δ *isolates are identified by initially selecting for co-transformation with a *TRP1 *plasmid and then replica-plating hundreds of these transformants to FOA medium [14]. This protocol uses many plates and is relatively work-intensive, so we asked whether substituting *URA3 *with the CORE-GS cassette, which includes the *Gal-SceI *fusion gene and the unique SceI site as well as *URA3 *(see Background), would improve the efficiency of targeted mutagenesis. We termed this modified version, "gsSSG mutagenesis".

Our initial experiments compared the frequency of FOA^R ^isolates in standard SSG and gsSSG mutagenesis. As a first step, we inserted either *URA3 *(standard SSG) or CORE-GS (gsSSG) into the same site in the genome (within the *IME1 *promoter) in the first transformation. These two strains were then separately transformed with the same three PCR fragments (Figure 1A, compare (i) and (ii)) along with the *TRP1 *plasmid. These fragments are all approximately 1 kb in length (Table 1, column 3), with one end from 90 - 200 bp from the site of marker insertion (column 4). We plated transformation mixtures on Trp^- ^medium, allowed Trp^+ ^colonies to grow, resuspended and pooled the colonies from an entire transformation plate, and then plated these cell suspensions on FOA (Table 1, column 5). The initial selection for the *TRP1 *plasmid is necessary for subsequent selection of FOA^R ^isolates [14]. We found that gsSSG mutagenesis yielded significantly more FOA^R ^isolates/Trp^+ ^cell than standard SSG mutagenesis (P = 0.03, paired t-test). Interestingly, for standard SSG, the FOA^R^/Trp^+ ^frequency varied 100-fold among these three fragments, whereas for gsSSG they varied only five-fold.

**Table 1:** f(FOA^R^ isolates) & recombinant fraction in SSG & gsSSG mutagenesis

PCR Fragment	Method	Frag. Size^a^	Distance Ins - End^b^	f(FOA^R^)^c^	FOA^R ^Tested^d^	Recomb. Fract (%)^e^
A	SSG	1.1	0.20	0.04	4	0
	gsSSG			1.3	5	100
B	SSG	1.0	0.12	0.0003	5	40
	gsSSG			1.9	6	100
C	SSG	1.0	0.09	0.004	4	25
	gsSSG			0.4	6	100

^a ^Size in kb of PCR fragment used in transformation

^b ^Distance in kb from marker insertion site to the fragment end nearest this site

^c ^Frequency of FOA^R ^isolates among Trp^+ ^isolates

^d ^Number of FOA^R ^isolates tested by diagnostic PCR for fragment-genome recombination, each row reports on a single transformation

^e ^Fraction of FOA^R ^isolates that derived from fragment-genome recombination

In theory, transformants can become FOA^R ^through a variety of mechanisms, including recombination with the PCR fragment (the desired result) or conversion of the *URA3 *allele by the genomic *ura3-1 *allele present in the strains used for this study. Thus, the efficacy of SSG mutagenesis is reflected both by the frequency of FOA^R ^isolates and by the fraction of these isolates resulting from replacement of the marker with the PCR fragment, termed "the recombinant fraction".

The recombinant fractions in standard SSG and gsSSG mutagenesis were determined by diagnostic PCR of genomic DNA isolated from FOA^R ^isolates, using primers flanking the targeted region (Table 1, column 6-7). Overall, the recombinant fraction for standard SSG mutagenesis was 23% (3/13), consistent with the results of our previous study, when 33% (2/6) of FOA^R ^transformants replaced the *URA3 *marker [14]. In contrast, in gsSSG mutagenesis, 100% (17/17) of the FOA^R ^transformants replaced the *URA3 *marker, significantly higher than the replacement efficiency in standard SSG mutagenesis (Fisher's exact test, P < 0.0001). These results suggest that the induction of the double-strand break in gsSSG mutagenesis significantly increases the recombinant fraction among FOA^R ^transformants relative to standard SSG mutagenesis.

The above analysis compared gsSSG and standard SSG for only three fragments. To investigate the parameters that affect the efficiency of this mutagenesis, we next transformed the *URA3 *strain with 46 different PCR fragments and the *CORE-GS *strain with 34 different PCR fragments. For both protocols, these fragments ranged in size from 0.4 - 1.1 kb, and the distance between the nearest end and the marker insertion site ranged from 4 - 477 bp, so we asked whether either of these parameters influenced the efficiency of marker replacement. For each fragment, 1 - 39 FOA^R ^transformants were tested by diagnostic PCR as above. For gsSSG mutagenesis, as with the three fragments tested previously, almost all of the FOA^R ^isolates derived from marker replacement (172/176 = 98%). In contrast, in standard SSG mutagenesis, the recombinant fraction varied from 0 - 100%, with the average being approximately 26%. To investigate the parameters that affected recombinant fraction in standard SSG mutagenesis, we analyzed the above data to determine if this variation correlated with the length of the fragment and/or the distance between the nearest end and the *URA3 *insertion site.

To determine whether the overall length of the PCR fragment affected standard SSG mutagenesis, we employed a logistic regression analysis (see Methods). From this analysis we found that the frequency of marker replacement did not significantly depend on the fragment length (P = 0.12).

The same logistic regression analysis measured the relationship between the recombinant fraction and the distance from the marker insertion site to the closest end of the fragment (Figure 1A). We found that the recombinant fraction did depend on this distance (P = 0.008). More specifically, the recombinant fraction increased as the distance increased. This dependence can be visualized graphically by considering only those fragments in which at least 6 transformants were analyzed so as to minimize sampling error (Figure 1B) or by the fitted logistic regression of all data, including those with limited replicas (Figure 1C, solid line).

Thus, gsSSG mutagenesis yields a much higher frequency of FOA^R ^isolates than standard SSG mutagenesis, and in gsSSG mutagenesis, unlike standard SSG mutagenesis, virtually every FOA^R ^isolate results from recombination between the PCR fragment and the genome, i.e. the recombinant fraction is close to 100%. The practical consequence of this much higher efficiency of marker replacement is that in gsSSG mutagenesis recombinants can be isolated by streaking colonies directly from transformation plates to FOA medium (see Methods). Because gsSSG mutagenesis does not require replica plating, it is much faster and requires far fewer FOA plates than standard SSG mutagenesis. For this reason, the remaining experiments in this study focus on the parameters affecting the efficiency of gsSSG.

### Effect of distance between mutation and CORE-GS insertion site on the frequency of incorporating mutations

**Figure 2:**
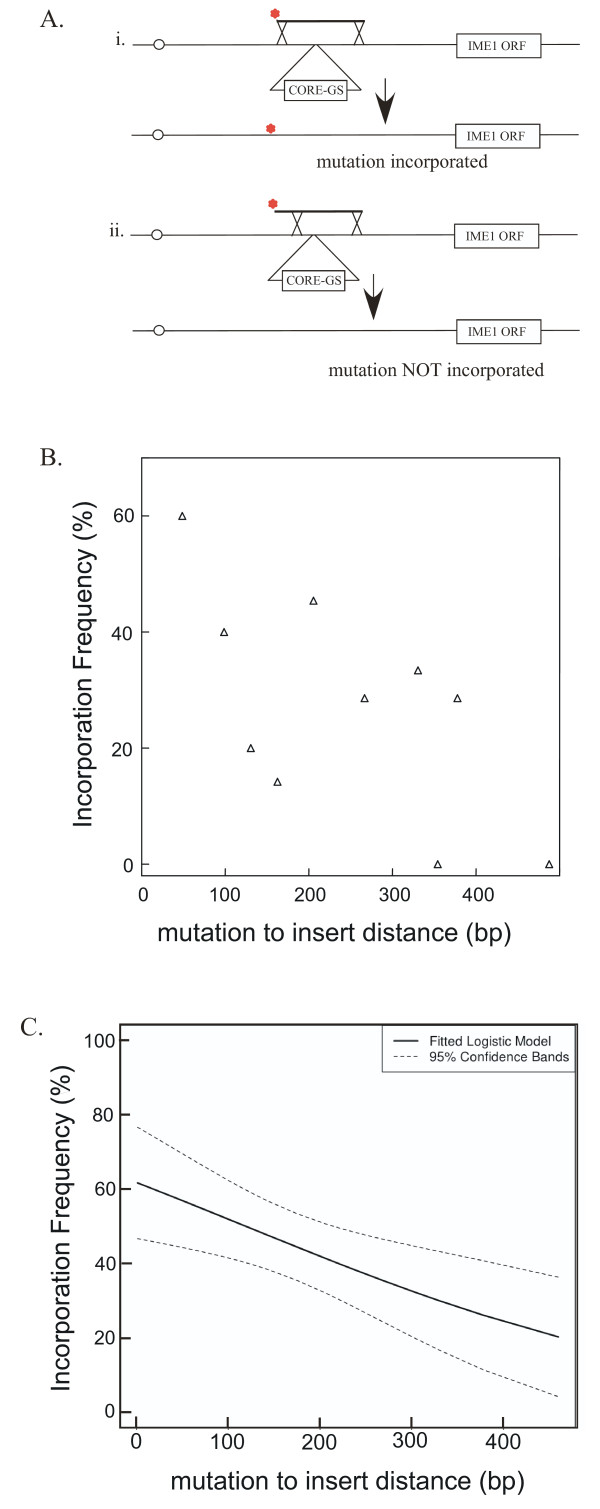
**Mutation incorporation depends on mutation-to-insertion site distance in gsSSG mutagenesis**. A) Recombination between a PCR fragment and the genome incorporates the mutation when the genetic exchange (crossover) occurs at the end of the fragment (i), but does not incorporate the mutation when the genetic exchange is in the central region of the fragment (ii). B) Relationship between mutation-to-insertion distance and mutation incorporation. The mutation incorporation frequency, defined as fraction of recombinants that have incorporated the targeted mutation, is shown for transformants that contained a mutation the indicated distance from the CORE-GS insertion site. Only transformations where > 5 recombinants were analyzed are included in this graph. C) Logistic regression analysis of the same data as in B) except that all data was included regardless of the number of recombinants analyzed for a given transformation. The solid line represents the fitted logistic model, and the dashed line represents 95% confidence bands around the model.

The success of gsSSG mutagenesis depends not only on marker replacement as discussed above but also on incorporation of the mutation present on one end of the fragment. Mutation incorporation and recombinant fraction are not equivalent because the PCR fragment can recombine at its end and incorporate the mutation (Figure 2A, i), or it can recombine in the region between the mutation and the marker insertion and not incorporate this mutation (Figure 2A, ii). We sequenced the targeted region in isolates from the 34 gsSSG transformations described above. For each transformation, only isolates shown to have replaced the marker were sequenced, and 1-11 such isolates were sequenced for each transformation. We found that the frequency of incorporating this mutation ranged from 0 - 100%. This broad range suggests that differences between the fragments affect mutation incorporation.

In principal, either the length of the fragment or the distance between the mutation and the CORE-GS insertion site (termed the "mutation-to-insert" distance) could influence incorporation frequency. In order to test these hypotheses, we analyzed this data by logistic regression analysis. As a first test, we examined the relationship between mutation incorporation and fragment length. As above when measuring recombinant fractions, the efficiency of incorporating the targeted mutation does not depend on the fragment length (P = 0.34). In contrast, we found that mutation incorporation did depend on the distance between the mutation and the insertion site, which ranged from 1 - 487 bp (P = 0.016). In particular, the frequency of mutation incorporation increased as this distance diminished. This effect can be visualized graphically by considering only transformants in which > 5 isolates were examined in order to minimize sampling errors (Figure 2B) and from the fitted logistic regression analysis of the complete data set (Figure 2C). As a final test of the effect of the distance of the mutation to the marker insertion site, we targeted a mutation 1.0 kb from the insertion site using a 1.1 kb PCR fragment. Of 9 FOA^R ^isolates tested, each had replaced the GS-CORE, but only one incorporated the targeted mutation. Thus, it is possible to introduce mutations at least 1.0 kb from the insertion site, but the incorporation frequency for these mutations is likely to be low compared to mutations much closer to the insertion site.

### Untargeted mutations

For gsSSG to be effective, in addition to incorporating the targeted mutation, the rest of the inserted fragment must be free of untargeted mutations. Untargeted mutations in SSG mutagenesis typically result from errors during amplification of the PCR fragment. Because errors can occur at any round of PCR, errors may accumulate at different locations in different molecules from the same PCR. Thus, the risk of introducing untargeted mutations cannot be eliminated by batch sequencing the population of fragments derived from a single PCR. Instead, the entire genomic region corresponding to the PCR fragment must be amplified and sequenced for each recombinant to determine if there were untargeted mutations introduced on the PCR fragment.

To estimate the frequency of untargeted mutations, we amplified and sequenced a portion of the targeted region of many different recombinants. When the PCR fragments used for transformation were synthesized with standard Taq polymerase, the frequency of untargeted mutations was 8.3 × 10^-4 ^mutations/bp for standard Taq (14.5 kb from 19 PCR fragments was analyzed). When these PCR fragments were instead synthesized using a high-fidelity polymerase (exTaq) only 4.5 × 10^-4 ^mutations/bp were observed (11.2 kb from 10 PCR fragments was analyzed). The probability of a transformant having no untargeted mutations (P_U_) can be estimated from the frequency of untargeted mutations (m) and the size of the fragment (N) using the following formula: P_U _= (1-m)^N^. Thus for a 0.5 kb fragment, P_U _= 66% using standard Taq polymerase and P_U _= 80% using exTaq. In contrast, for a 1.0 kb fragment, P_U _= 44% using standard Taq polymerase and P_U _= 64% using exTaq. Thus, the probability of transformants having no untargeted mutations is considerably increased both by using high-fidelity Taq polymerases and by minimizing the size of the PCR fragment. Note that for a particular targeted mutation and insertion site, limiting the distance between the unmutated end and the marker insertion site can minimize the fragment size without decreasing the efficiency of marker replacement.

## Discussion

The principal results reported in this study are as follows. First, substituting the CORE-GS cassette for *URA3 *in SSG mutagenesis greatly increased the utility of the method. Second, the efficiency of incorporating the targeted mutation does not depend on the overall fragment length in the range from 0.4 - 1.1 kb but does increase as the distance of the mutation to the site of the CORE-GS insertion decreases. Finally, strategies minimizing the number of untargeted mutations can substantially increase the overall success of gsSSG. Below we discuss the implications of these results and discuss the advantages and disadvantages of gsSSG relative to other methods for targeting precise mutations into *S. cerevisiae*.

In both gsSSG and standard SSG, a marker is inserted in the genome in a first transformation and then replaced with a PCR fragment in a second transformation. However, in two respects this second transformation is much more efficient in gsSSG mutagenesis than in standard SSG mutagenesis. First, the frequency of FOA^R ^isolates/cfu was much higher in gsSSG than in standard SSG. Second, the fraction of FOA^R ^isolates that derived from fragment/genome recombination (i.e. the recombinant fraction) was much higher in gsSSG mutagenesis than in standard SSG mutagenesis. Indeed, in gsSSG mutagenesis, among 34 different PCR fragments introduced into the genome, all but one inserted with 100% efficiency. In contrast, in standard SSG mutagenesis this recombinant fraction depended strongly on the distance between marker insertion site and the closest fragment end-- the greater the distance, the higher the recombinant fraction. Thus, the CORE-GS cassette, developed to increase the efficiency of the *delitto perfetto *protocol [12,13], also dramatically improves SSG mutagenesis. For this reason, it is likely that the CORE-GS will also improve the efficiency of the several modifications of SSG described previously: namely, replacing alleles (asSSG), inserting (iSSG) or deleting (dSSG) sequences, or introducing mutations at random within a defined region of the genome (RDL mutagenesis) [14,15].

The higher recombinant fraction in gsSSG relative to standard SSG as well as the lower dependence on insertion site - fragment end distance in the former method is likely explained by the SceI-directed double-strand break introduced during gsSSG. Double-strand breaks greatly stimulate most types of homologous recombination in yeast (reviewed in [16,17]. In contrast, in the absence of this targeted break (i.e. in standard SSG mutagenesis), the length of homology between the nearest end and the break becomes limiting for recombination. For example, the length of homology may limit the stability of a recombination intermediate in standard SSG, whereas the induced double-strand break in gsSSG could result in a more stable recombination intermediate that does not depend on a long region of homology. In this respect, it is worth noting that the recombinant fraction in standard SSG did not depend significantly on the overall length of the fragment, though we tested fragments ranging from 0.4 - 1.1 kb, so a longer region of homology on one side of the insertion may not effectively compensate for a shorter region of homology on the other side.

The practical consequence of the greater recombination frequency in gsSSG mutagenesis relative to standard SSG mutagenesis is that it is much easier to identify recombinants in gsSSG mutagenesis. For example, in standard SSG mutagenesis, it is necessary to replica plate hundreds of transformants to identify a sufficient number of FOA^R ^isolates that have replaced the marker with the PCR fragment. In contrast, in gsSSG mutagenesis a portion of a transformation plate can be scraped using a single toothpick and struck on FOA, and this streak will almost always contain only recombinants. Because gsSSG mutagenesis does not require replica plating, it uses many fewer FOA plates and is more cost-effective and faster than the standard SSG mutagenesis protocol.

Although FOA^R ^isolates generated by gsSSG mutagenesis almost always result from replacement of the CORE-GS cassette with the PCR fragment, this replacement does not always result in the incorporation of the mutation. In particular, when the genetic exchange occurs between the mutation and the cassette insertion site, the cassette is replaced with a portion of the PCR fragment that does not include the mutation (Figure 2A, ii). We found that the greater the distance between the mutation and the cassette insertion, the lower the probability of incorporating the mutation. This effect is likely a manifestation of the classic relationship between distance and recombination frequency; i.e., the larger the region, the greater probability that it contains a recombination event. As one caveat, it is not possible to extrapolate from our data to distances much greater than the range tested; for example, DNA ends are thought to be recombinogenic, so genetic exchange at these ends incorporating the mutation may be relatively efficient even at longer distances.

A final parameter affecting the success of gsSSG mutagenesis is fragment size. Although overall fragment size in the size range from 0.4 -1.1 kb has little or no effect on either the recombinant fraction or mutation incorporation, the fragment size affects the overall success of gsSSG because longer fragments have an increased chance of incorporating untargeted mutations. For gsSSG to be successful, the final product must contain only the targeted mutation and no other mutations. Because gsSSG depends on incorporating a PCR fragment into the genome, and untargeted mutations may be present on this fragment, it is essential to sequence the entire region of the resulting mutant corresponding to this fragment. By using a high-fidelity thermophilic polymerase to construct the fragments, and by minimizing the overall size of the PCR fragment, the number of transformants that need to be sequenced can be reduced.

We define the expected success rate of gsSSG mutagenesis (S) as the probability that an FOA^R ^isolate contains the targeted mutation and no other mutations. S can be calculated as follows: S = P_U _× P_M_, where P_U _= frequency of isolates that lack untargeted mutations (as described in Results) and P_M _= frequency of isolates that contain the targeted mutation. P_M _can be estimated from Figure 2C based on the distance of the mutation from the marker insertion site. In this study, the relationship between distance and mutation incorporation was determined at a single locus. It remains to be seen if this relationship is similar throughout the genome. In general, the frequency of homologous recombination in *S. cerevisiae *(including fragment/genome recombination) varies considerably between loci [18-20]. However, marker replacement during standard SSG mutagenesis has been shown to occur efficiently at several different sites in the genome [15], and the same is true for *delitto perfetto *[12].

Calculating expected success rate (S) is useful for estimating the number of recombinants (x) that should be sequenced to have a given probability of identifying a precise mutation. For example, consider a 0.5 kb PCR fragment synthesized with exTaq in which the mutation is 200 bp from the CORE-GS site. Since P_M _= 0.42 (based on Figure 2C), m = 4.5 × 10^-4 ^for this polymerase, and N = 500, then S = [(1 - (4.5 × 10^-4^))^500^] × (0.42) = 0.34. Thus, in this example to have a > 90% chance of identifying at least one isolate containing only the targeted mutation, we calculate that 0.90 < (1-S)^x^, or x = log (1-S)/log (0.90). By rearranging this equation, x < log (1 - 0.34)/log (0.9) = 4, meaning that if 4 FOA^R ^isolates are sequenced, there is a >90% chance at least one isolate will contain the targeted mutation and no other mutations.

*Delitto perfetto*, MIRAGE, and gsSSG mutagenesis achieve the same aim: efficiently targeting mutations to the yeast genome, but each method has different advantages. An advantage of both *delitto perfetto *and MIRAGE is that because the amount of homologous DNA introduced into the genome is limited to the size of a primer or oligonucleotide, the number of untargeted mutations for these methods will likely be lower than in gsSSG mutagenesis-- where an entire PCR fragment is introduced into the genome. Relative to both *delitto perfetto *and gsSSG mutagenesis, MIRAGE requires only a single transformation, but on the other hand, MIRAGE requires the extra step of ligating PCR fragments. *Delitto perfetto *has an advantage over MIRAGE when the goal is to generate multiple mutants each with a different mutation within a defined region of the genome; this is because once the Gal-SceI CORE has been inserted into the genome, mutations can be introduced anywhere within a 100-200 bp of the CORE site simply by using different oligonucleotides in the second step. Similarly, the principal advantage of gsSSG mutagenesis over the other two methods is that mutations may be targeted at least 460 base pairs on either side of the insertion by simply synthesizing different PCR fragments. In summary, MIRAGE may be the most efficient method for introducing a single point mutation in the genome, *delitto perfetto *the most efficient method when an array of mutants are desired each with a different mutation within a 200 bp region and gsSSG mutagenesis the most efficient method when an array of mutants are desired each with a different mutation within an at least 900 bp region.

## Conclusions

Modification of the SSG mutagenesis protocol to incorporate the CORE-GS marker/endonuclease module greatly improves the efficiency of targeting precise mutations. The probability of incorporating the targeted mutation in gsSSG mutagenesis decreases as the distance between the insertion site and the targeted mutation increases but remains efficient at least 460 bp on either side of the insertion site. Minimizing the size of the PCR fragment used in gsSSG increases the success rate because small fragments show the same high rate of recombination with the genome as do larger fragments and at the same time have a lower probability of containing untargeted mutations. Thus, gsSSG adds another powerful method to the tools available to the yeast geneticist.

## Methods

### Yeast strains

All SSG and gsSSG mutagenesis was performed on strains of the W303 background. For Step 1 of SSG mutagenesis, a 1.1 kb PCR fragment containing *URA3 *was inserted 1131 bp upstream of the *IME1 *start codon in SH773 to generate strain SH2608 (*MATα ade2 can1:ADE2:CAN1 his3-11,15 leu2-3,112 trp1-3'Δ ura3-1 prIME1::URA3*) or was inserted at the same position into SH3650 to yield SH4132 (*MATα ade2 can1:ADE2:CAN1 his3-11,15 leu2-3,112 trp1-3'Δ ura3-1 rme1Δ::LEU2 prIME1-lacZ::URA3*). For Step 1 of gsSSG mutagenesis, a 4.6 kb PCR fragment containing the CORE-GS [13] was inserted at the same position as above into SH3650 to generate SH4200 (*MATα ade2 can1:ADE2:CAN1 his3-11,15 leu2-3,112 trp1-3'Δ ura3-1 rme1Δ::LEU2 prIME1-LacZ::CORE-GS*). Both insertions were targeted based on 40 bp on either end of the PCR fragment homologous to the 40 bp flanking either side of the insertion site.

### PCR and sequence analysis

Amplification of PCR fragments for transformation, to verify loss of CORE-GS, and to amplify genomic DNA for sequencing was as described previously [14] and utilized either Taq or exTaq (TaKaRa). The PCR fragment to introduce *URA3 *into the *IME1 *promoter for step 1 of SSG mutagenesis was amplified using the following 60-mer primers:

GTGCGTATCTTTGTTTACTTTTCGTCTTCGAGGGGAAGGAtaactatgcggcatcagagc and ATCCTGGCGCCCCCTCCTGCGGGCACGCATGCGCCTTTGAcctgatgcggtattttctcc, and the plasmid, RS306 [21]. The fragment to introduce *CORE-GS *into the *IME1 *promoter for step 1 of gsSSG was amplified using the following 60-mer and 80-mer primers:

GTGCGTATCTTTGTTTACTTTTCGTCTTCGAGGGGAAGGAttcgtacgctgcaggtcgac and ATCCTGGCGCCCCCTCCTGCGGGCACGCATGCGCCTTTGATAGGGATAACAGGGTAATttggatggacgcaaagaagt and the template plasmid, pGSKU. For both sets of primers, lower case letters represent nucleotides homologous to the vector sequence, the underlined nucleotides are the SceI site, and the remaining nucleotides are homologous to the target site. To introduce mutations in the second step of SSG and gsSSG, a fragment was amplified using a 20-mer primer, a 40-mer primer containing a single point mutation in the central 20 bp, and the template plasmid, pS240. To detect marker replacement in standard SSG we used the following primers: CCGAAAACGTACGGCTAACT and AACGTTGTAAACGCAATCACC. These primers yielded a 2.7 kb fragment when the markers were present and a 1.5 kb fragment when the markers were replaced. To detect marker replacement in gsSSG we used a 5' primer, CCGTACAGCTATCGTTTCAGG, together with two 3' primers, TAGTCCCTTTGCAGACATG and TCGCCTTTGTCGTCTAAACC. These primers yielded a 1.7 kb fragment when the markers were present and a 0.7 kb fragment when the markers were replaced. To detect targeted and untargeted mutations in the genome after transformation, we amplified and sequenced a fragment from the genome that contained the entire region corresponding to the introduced PCR fragment.

### SSG and gsSSG mutagenesis

SSG mutagenesis was performed as describe previously [14]. The first step of gsSSG (introducing the CORE-GS into the yeast genome) was performed using a standard protocol for marker gene insertion [14]. The second step of gsSSG mutagenesis (replacement of the marker with a PCR fragment of the genome containing a mutation) was performed as follows: Prior to transformation, overnight cultures of SH2608 or SH4200 were inoculated at 5 × 10^6 ^cells/ml in YPGal medium and grown for approximately 6 hrs to reach 2 × 10^7 ^cells/ml. PCR fragments (100-200 ng) were transformed into SH2608 or SH4200 together with a vector carrying *TRP1*^+^, pTV3 (1 μg), using a standard LiOAc protocol [22] with a 40 minute heat-shock at 44°. Enough of the transformation was spread on Trp^- ^medium so that 5,000-10,000 colonies formed. A region of the transformation plate containing several hundred colonies (approximately 1/20 of plate) was scraped on to a single toothpick and then streaked to one quadrant of an FOA plate. By only scraping non-overlapping regions from the transformation plate, we ensured that FOA^R ^colonies from different streaks derived from independent transformants. Typically, we tested 4-12 independent FOA^R ^colonies for each transformation.

### Statistical analysis

Because the results from the multiple transformation experiments represented in Figure 1C and Figure 2C were either success (recombination or mutation incorporation) or failure (no recombination or no mutation incorporation), logistic regression analysis was performed on these data. For this purpose, we used the SAS and R software packages. For the data in Figure 1C the following possible predictor variables were employed: 1) size of fragment, and 2) distance of the closest fragment end to site of marker insertion. For the data in Figure 2C, the following possible predictor variables were employed: 1) size of fragment, and 2) distance of mutation to site of marker insertion. The Akaike Information Criterion (AIC) was used as a tool in model selection. All data used for these analyses are presented in Additional Files: recombinant fraction from standard SSG mutagenesis (Additional file 1, Table S1), recombinant fraction for gsSSG mutagenesis (Additional file 2, Table S2), and mutation incorporation for gsSSG mutagenesis (Additional file 3, Table S3).

## Authors' contributions

SP and HLW carried out the experiments for these studies and contributed to the design of the study. TJF performed the statistical analysis of the data. SMH conceived of the study, contributed to the design and analysis of results and prepared the manuscript. All authors read and approved the final manuscript.

## Supplementary Material

Additional file 1 **Fragment size, distance between near end and marker insertion site, and efficiency of marker replacement for standard SSG mutagenesis**. This Excel file lists the efficiency of marker replacement (i.e. the recombinant fraction) among total FOA^R ^isolates for each targeted transformation using standard SSG mutagenesis. Column A shows the strain transformed with a PCR fragment containing a single mutation. Column B shows the position of this mutation; position 1 is 2000 bp upstream of the *IME1 *start codon. Column C shows the distance of the nearest end of the PCR fragment to the *URA3 *insertion. Column D shows the size of the PCR fragment. Column E shows the number of FOA^R ^isolates tested. Column F shows the percentage of these isolates that have replaced the marker as determined by PCR (the recombinant fraction). Column G shows the result for each isolate in the order they were tested, where a "1" represents marker replacement and a "0" represents no replacement. Click here for file

Additional file 2 **Fragment size, distance between near end and marker insertion site, and efficiency of marker replacement forgsSSG mutagenesis**. This Excel file lists the efficiency of marker replacement (i.e. the recombinant fraction) among total FOA^R ^isolates for each targeted transformation using gsSSG mutagenesis. Column A shows the strain transformed with a PCR fragment containing a single mutation. Column B shows the position of this mutation as in Table S1. Column C shows the distance of the nearest end of the PCR fragment to the CORE-GS insertion. Column D shows the size of the PCR fragment. Column E shows the number of FOA^R ^isolates tested. Column F shows the percentage of these isolates that have replaced the marker as determined by PCR (the recombinant fraction). Column G shows the result for each isolate in the order they were tested, where a "1" represents marker replacement and a "0" represents no replacement. Click here for file

Additional file 3 **Fragment size, distance between mutation and marker insertion site, and efficiency of mutation incorporation for gsSSG mutagenesis**. This Excel file lists the efficiency of incorporating the targeted mutation among total FOA^R ^isolates for each targeted transformation using gsSSG mutagenesis. Column A shows the strain transformed with a PCR fragment containing a single mutation. Column B shows the position of this mutation as in Table S1. Column C shows the distance of the mutation to the CORE-GS insertion. Column D shows the size of the PCR fragment. Column E shows the number of isolates containing a replaced marker that were sequenced. Column F shows the percentage of these isolates that have incorporated the mutation based on two-strand sequence. Column G shows the result for each isolate in the order they were tested, where a "1" represents incorporation and a "0" represents no incorporation. Click here for file
